# Intravenous thrombolysis or mechanical thrombectomy do not increase risk of acute symptomatic seizures in patients with ischemic stroke

**DOI:** 10.1038/s41598-020-78012-y

**Published:** 2020-12-03

**Authors:** Johann Philipp Zöllner, Björn Misselwitz, Thomas Mauroschat, Christian Roth, Helmuth Steinmetz, Felix Rosenow, Adam Strzelczyk

**Affiliations:** 1 Department of Neurology, Epilepsy Center Frankfurt Rhine-Main, Center of Neurology and Neurosurgery, Goethe University Frankfurt, Schleusenweg 2-16, 60528 Frankfurt am Main, Germany; 2 LOEWE Center for Personalized Translational Epilepsy Research (CePTER), Goethe University Frankfurt, Frankfurt am Main, Germany; 3 Quality Assurance Office Hessen (GQH, Geschäftsstelle Qualitätssicherung Hessen), Eschborn, Germany; 4 Department of Neurology and Epilepsy Center Hessen, Philipps-University Marburg, Marburg (Lahn), Germany; 5 Department of Neurology, DRK-Kliniken Nordhessen, Kassel, Germany

**Keywords:** Cerebrovascular disorders, Epilepsy, Stroke

## Abstract

Recent data have suggested that performing recanalizing therapies in ischemic stroke might lead to an increased risk of acute symptomatic seizures. This applies to both intravenous thrombolysis and mechanical thrombectomy. We therefore determined the frequency of acute symptomatic seizures attributable to these two recanalization therapies using a large, population-based stroke registry in Central Europe. We performed two matched 1:1 case–control analyses. In both analyses, patients were matched for age, stroke severity on admission and pre-stroke functional status. The first analysis compared patients treated with intravenous thrombolysis to a non-recanalization control group. To isolate the effect of mechanical thrombectomy, we compared patients with both mechanical thrombectomy and intravenous thrombolysis to those with only intravenous thrombolysis treatment in a second analysis. From 135,117 patients in the database, 13,356 patients treated with only intravenous thrombolysis, and 1013 patients treated with both intravenous thrombolysis and mechanical thrombectomy were each matched to an equivalent number of controls. Patients with intravenous thrombolysis did not suffer from clinically apparent acute symptomatic seizures significantly more often than non-recanalized patients (treatment = 199; 1.5% vs. control = 237; 1.8%, *p* = 0.07). Mechanical thrombectomy in addition to intravenous thrombolysis also was not associated with an increased risk of acute symptomatic seizures, as the same number of patients suffered from seizures in the treatment and control group (both n = 17; 1.7%, *p* = 1). In a large population-based stroke registry, the frequency of clinically apparent acute symptomatic seizures was not increased in patients who received either intravenous thrombolysis alone or in conjunction with mechanical thrombectomy.

## Introduction

Acute symptomatic seizures are a serious complication after an ischemic stroke, and are associated with increased mortality and diminished functional outcomes^1–4^. Important risk factors for their occurrence are the severity of the stroke, cortical involvement, and hemorrhagic transformation, but also non-neurological disorders such as systemic infections^1,5–11^. Acute symptomatic seizures by definition occur within seven days after stroke onset^12^. Seizures occurring later than that lead to a diagnosis of structural epilepsy, if a structural lesion due to the stroke is apparent using brain imaging^1^. Ischemic stroke is a major risk factor for the development of structural epilepsy^13^, and in older patients ischemic stroke is the most common cause of structural epilepsy^14,15^.

The implementation of recanalization procedures such as intravenous thrombolysis (IVT) with recombinant tissue plasminogen activator (rt-PA) within a time window of 4.5 h after symptom onset, and mechanical thrombectomy (MT) in selected cases, has dramatically improved functional outcomes in stroke patients, significantly reduced their mortality, and has thus become standard of care^16–22^.

Recently, a number of publications have suggested that recanalization attempts after ischemic stroke with either IVT or MT could increase the risk of acute symptomatic seizures or subsequent epilepsy^23–25^. However, the data supporting this notion are from studies in which a limited number of patients suffered from acute seizures, and other reports could not show a correlation between recanalization of an arterial cerebral vessel and the occurrence of acute symptomatic seizures^26–30^. In addition, the vast majority of these studies only investigated IVT therapy, reporting a risk for acute symptomatic seizures after rt-PA treatment between 1–4%. Only a few studies have investigated the effect of MT on the frequency of acute symptomatic seizures^23,31–33^.

Here we evaluated whether IVT or MT in conjunction with IVT were associated with an increased risk of acute symptomatic seizures in a large population-based dataset from Central Europe. Reporting results from large datasets can significantly increase the knowledge base and thus inform the ongoing debate on treatment-related acute symptomatic seizures in ischemic stroke.

## Methods

For this matched case–control analysis, we retrospectively evaluated data from the stroke registry of the Quality Assurance Office Hessen (Geschäftsstelle Qualitätssicherung Hessen [GQH], Eschborn, Germany; www.gqhnet.de) over a 13-year period (2004–2016). The purpose of the GQH stroke registry is to assess the quality of treatment in patients with strokes at more than 100 hospitals in Hessen, a federal state of Germany with more than six million inhabitants (www.destatis.de; 6,176,172 inhabitants on December 31, 2015). Either the treating physician or a coding specialist enters the patient data. Data entry is mandatory by law for all hospitals in Hessen, and cross-validation with billing data according to §21 Krankenhausentgeltgesetz (the “Hospital Remuneration Act”) shows more than 99% completeness. Data used in this study is available from the GQH, pending certain prerequisites.

We included adult patients who suffered from ischemic strokes (Code I63.x; International Statistical Classification of Diseases and Related Health Problems, 10th revision) and who were admitted to hospital within 24 h after symptom onset. Stroke was defined as an acute loss of neurological function due to a cerebrovascular cause persisting for more than 24 h, with exclusion of intracerebral hemorrhage^34^ using adequate imaging (e.g., cranial computed tomography or cranial magnetic resonance imaging) or imaging findings of ischemic stroke.

We collected demographic data (age and gender), clinical data (National Institutes of Health Stroke Scale [NIHSS] score at admission and modified Rankin Scale [mRS] at admission), and vascular risk-factor data (arterial hypertension, diabetes mellitus, hypercholesterolemia, previous stroke). Epileptic seizures were entered into the database by treating physicians when they were assumed to be stroke complications, but no semiological seizure details were collected. Similarly, the database does not include data on hemorrhagic transformation or detailed cortical-stroke localization.

We performed two separate matched case–control analyses. For the first analysis, we matched patients who underwent thrombolysis (IVT+) 1:1 to those without any recanalization therapy (IVT−) based on age, the pre-stroke mRS, and the NIHSS at admission. For the second analysis, we matched patients who received IVT followed by mechanical thrombectomy (IVT+MT+) 1:1 to a historical cohort of patients who solely underwent thrombolysis (IVT+MT−) according to the same parameters as in the first analysis. We did not match patients with only MT (and no IVT) due to a limited number of patients, and clinical disorders that may have precluded prior IVT and thus might bias the analysis. The historical cohort of IVT+MT− patients was taken from the years 2004–2011, before the use of MT was established. We chose the matching parameters for their relevance to the clinical decision to attempt either a pharmacological or a mechanical recanalization, which commonly depends *inter alia* on the severity of the stroke and pre-stroke functional status.

Matching of patients in both analyses was achieved using the FUZZY matching function implemented in the software SPSS (v.26, IBM Corp., Armonk, NY, USA) with the following constraints: age ± 2 years and NIHSS ± 2 score points and no flexibility on pre-stroke mRS score. Descriptive statistics were calculated using the Chi-Squared test for nominal variables and the Mann–Whitney U test for ordinal or continuous variables. We performed no additional adjustment of analyses for baseline differences after the matched control selection. A *p* value < 0.05 (two-sided) was considered statistically significant. All statistical analyses were performed using SPSS.

### Ethics approval

All methods were carried out in accordance with relevant guidelines and regulations. The analysis was approved by the ethics committee of the Goethe-University Frankfurt. Informed consent was waived due to the retrospective nature of the study.

## Results

**Figure 1:**
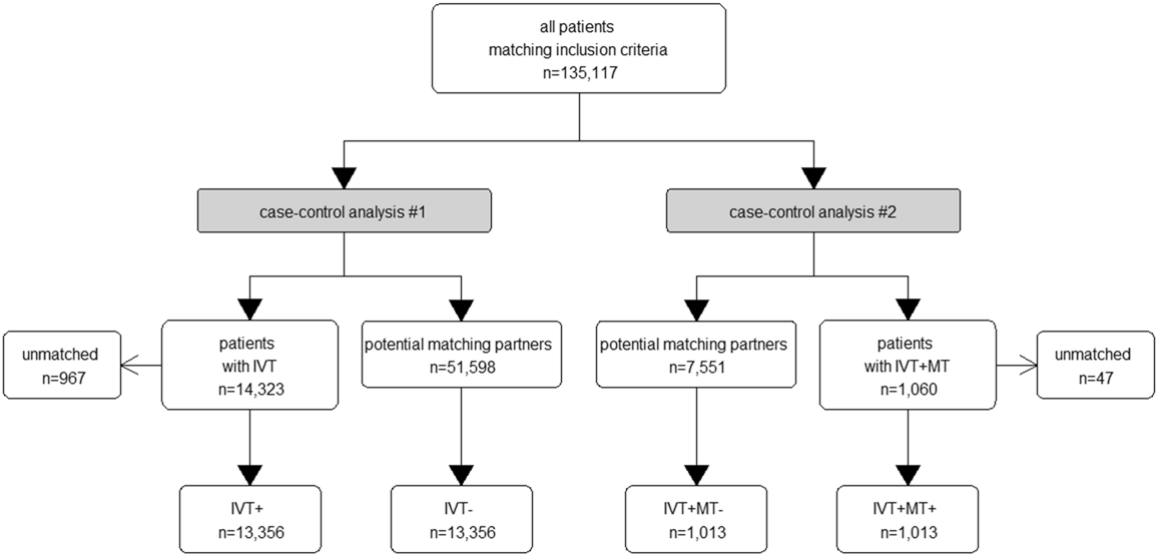
Flowchart showing the number of patients in the different cohorts. IVT, intravenous thrombolysis; MT, mechanical thrombectomy; IVT+, intravenous thrombolysis only; IVT−, no intravenous thrombolysis and no other recanalization attempt; IVT+MT−, intravenous thrombolysis, no mechanical thrombectomy; IVT+MT+, intravenous thrombolysis and mechanical thrombectomy.

Overall, we included 135,117 adult patients with an acute ischemic stroke who were admitted to hospital 24 h (at the latest) after stroke. Of these, 15,383 patients were either treated with IVT only (IVT+, n = 14,323) or additionally received MT (IVT+MT+, n = 1060) (Fig. 1).

We matched 13,356 patients treated with intravenous thrombolysis (IVT+) to 13,356 patients without thrombolysis treatment (IVT−) and 1013 patients with both thrombolysis and thrombectomy treatments (IVT+MT+) to 1013 patients with thrombolysis treatment only (IVT+MT−). In 536 patients, only MT was performed without prior IVT.

**Figure 2:**
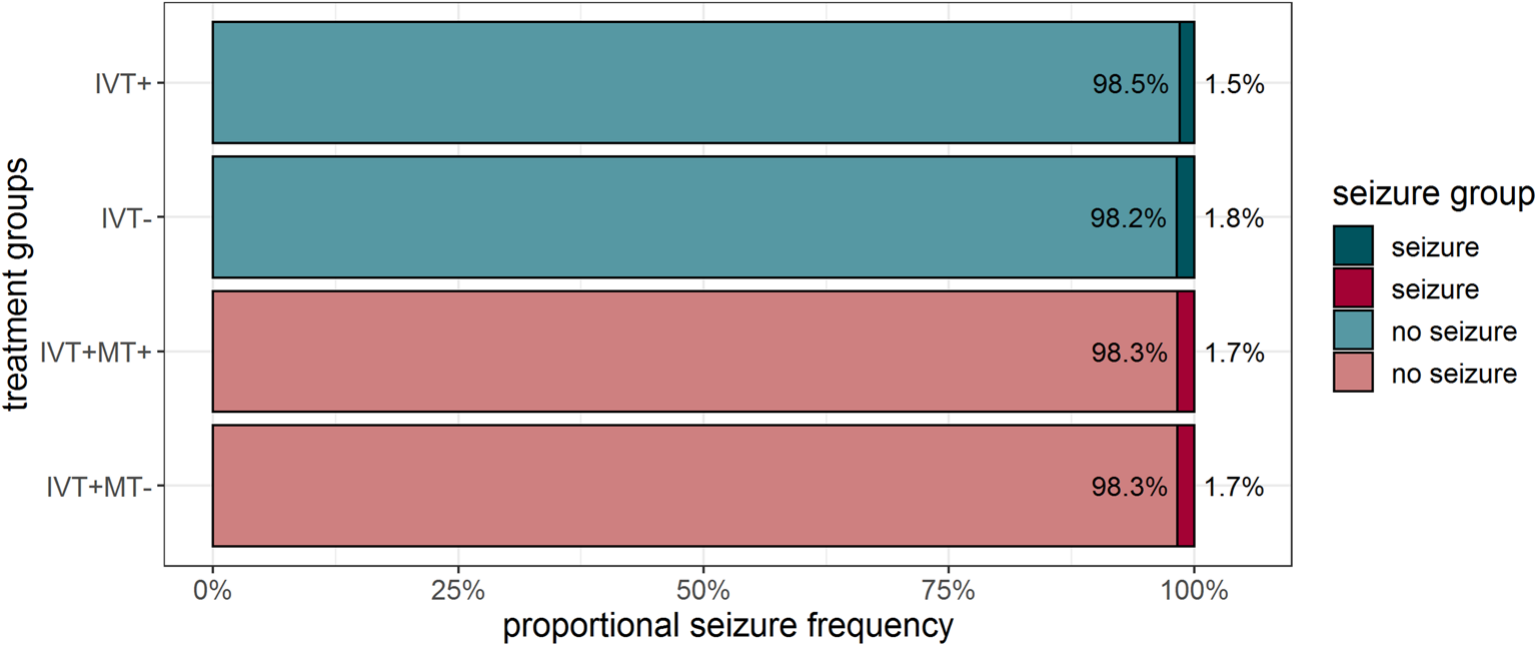
Proportional seizure frequency in the different treatment groups. IVT, intravenous thrombolysis; MT, mechanical thrombectomy; IVT+, intravenous thrombolysis only; IVT−, no intravenous thrombolysis and no other recanalization attempt; IVT+MT−, intravenous thrombolysis, no mechanical thrombectomy; IVT+MT+, intravenous thrombolysis and mechanical thrombectomy.

IVT with rt-PA did not increase the risk of acute symptomatic seizures. Of the 13,356 matched IVT+ patients, 199 (1.5%) suffered an acute symptomatic seizure compared to 237 (1.8%) patients who suffered seizures in the IVT− control group (*p* = 0.07). MT also did not increase the risk of acute symptomatic seizures. Of the 1,013 matched IVT+MT+ patients, 17 (1.7%) had an acute symptomatic seizure, the same number as in the IVT+MT− control group (*p* = 1) (Fig. 2).

Acute symptomatic seizures were also observed in 11 patients (2.1%) that underwent only MT without prior IVT. There was no significant difference compared to the 1.7% seizure frequency of the IVT+MT+ population (*p* = 0.745).

Clinical and demographic characteristics did not differ significantly between patients with seizures in the matched IVT+ and IVT− groups (see Table 1).

**Table 1:** Demographic and clinical characteristics of the patients that suffered from acute symptomatic seizures in the different treatment groups.

	IVT+ with seizures (n = 199)	IVT− with seizures (n = 237)	*p*	% Valid records
**IVT+ versus IVT− patients**				
Acute symptomatic seizure frequency	1.5%	1.8%	0.07	100
Age (SD)	75.8 (11.6)	73.6 (12.1)	0.05	100
Female gender	102 (51.5%)	122 (51.3%)	1.0	100
Mean NIHSS at admission (SD)	9.2 (6.3)	8.8 (6.5)	** < 0.001**	96
Median mRS before admission (range)	0 (0–5)	0 (0–5)	0.75	96.1
Arterial hypertension	176 (94.1%)	199 (90.9%)	0.22	93.1
Diabetes mellitus	49 (42.2%)	58 (41.1%)	0.86	58.9
Previous stroke	45 (41.6%)	70 (49.0%)	0.25	57.6
Non-neurological infection	28 (38.9%)	34 (39.1%)	0.98	36.5

	IVT+MT+ with seizures (n = 17)	IVT+MT− with seizures (n = 17)	*p*	% Valid records
**IVT+MT+ versus IVT+MT− patients**				
Acute symptomatic seizure frequency	1.7%	1.7%	1	100
Age (SD)	73.7 (10.5)	71.4 (12.7)	0.55	100
Female gender	8 (47.1%)	11 (64.7%)	0.30	100
Mean NIHSS at admission (SD)	14.1 (6.4)	13.8 (6.4)	0.407	100
Median mRS before admission (range)	0 (0–3)	0 (0–4)	0.12	100
Arterial hypertension	14 (93.3%)	14 (87.5%)	0.58	91.2
Diabetes mellitus	6 (87.5%)	2 (14.3%)	** < 0.001**	61.8
Previous stroke	1 (50%)	2 (16.7%)	0.29	58.8
Non-neurological infection	7 (58.3%)	0	**0.04**	47.1

Bold values indicate significant findings.

IVT+, patients from group with intravenous thrombolysis; IVT−, patients from matched group without any recanalization procedures; IVT+MT+, patients from group with intravenous thrombolysis and mechanical thrombectomy; IVT+MT−, patients from matched group with intravenous thrombolysis and without mechanical thrombectomy; mRS, modified Rankin Scale; SD, standard deviation.

In the IVT+MT+group, patients with seizures were significantly more likely to suffer from diabetes mellitus (*p* < 0.001) and significantly had more non-neurological infections (*p* = 0.04) compared to matched controls. Mean length of stroke-unit stay in patients with seizures did not differ between the IVT-only group and matched cases, with 4.9 days (SD 5.5) for the IVT+ group and 5.8 days (SD 8.7) for the IVT− group (*p* = 0.963). Mean length of stroke-unit stay was significantly longer in patients with seizures within the IVT+MT+ group at 11.4 days (SD 14.5) compared to 3.6 days (SD 3.4) in the seizure patients within the IVT+MT− group (*p* < 0.001).

There were 967 IVT+ patients without matching IVT− partners, and 47 IVT+MT+ patients without matching IVT+MT− partners (see Fig. 1). This was due to significantly younger age in the unmatched IVT+ patients versus the IVT− group [mean age 60.7 vs. 73.8 years (SD 14.3 and 12.8, respectively), *p* < 0.001] and more severe strokes as indicated by NIHSS at admission [median NIHSS 16 vs. 8 score points (mean 16.8 vs. 9.2), *p* < 0.001]. Likewise, the 47 unmatched patients in the IVT+MT+ group were significantly younger than the matched IVT+MT+ patients [mean age 65.0 vs. 69.8 years (SD 14.8 and 13.4, respectively), *p* = 0.018] and they had significantly higher stroke severity [median NIHSS 23 vs. 14 score points (mean 23.9 vs. 14.8), *p* < 0.001].

## Discussion

IVT and MT are paramount for improving patient outcomes and reducing mortality and thus represent the standard of care in ischemic stroke, if applicable^17,20^. Recently, an increase in acute symptomatic seizures has been suggested to be a complication of both recanalization methods^23–25^. Therefore, we evaluated the therapy-associated frequency of this potentially severe complication in a large retrospective case-controlled study of ischemic stroke patients.

Our results show that both IVT and MT were not associated with an increase in the frequency of acute symptomatic seizures after ischemic stroke. Acute symptomatic seizure frequencies were not significantly higher in patients receiving IVT, compared to not specifically treated patients and also not higher in those receiving IVT and MT when compared to a historical control of IVT-treated patients with similar demographic and clinical profiles on hospital admission. While the frequency of acute symptomatic seizures in the IVT+MT+ and matched control group was identical, the seizure frequency in the IVT-only patients was numerically even lower than in matched controls, although this did not reach statistical significance (*p* = 0.07).

Frequencies of acute symptomatic seizures noted by us are similar to those in several other studies in patients who underwent recanalization treatments, ranging between 1.2 and 3.8%^25,29,31,32^, with only two studies that reported higher frequencies between 8.1 and 23.4%^26,30^. These discrepancies are probably due to differences in seizure detection, classification and a referral bias in single-center studies with populations from tertiary stroke-centers. While it is known that standard electroencephalographic measurements significantly increase the likelihood of detecting post-stroke seizures^35^, only one study employed routine use of electroencephalography^28^. However, this prospective study did not report an overall seizure frequency^28^.

To date, several studies have found an increased risk for acute symptomatic seizures after IVT, one of them even after controlling for stroke severity^24,25^, and one study found an increased frequency of post-stroke epilepsy in those who underwent mechanical recanalization efforts^23^. The overall numbers of thrombolysed patients suffering from seizures in these studies (n = 12, n = 53 and n = 38, respectively) were smaller than in our population-based analysis. Both studies that found an increased risk for acute symptomatic seizures compared patients with seizures to either a matched^24^ or unmatched^25^ cohort of patients without seizures. In contrast to these studies, we compared patients with recanalization to those without the specific therapy in question. This allowed us to better identify the specific effect of recanalization therapy on acute seizure frequency. Importantly, our chosen matching parameters reflected information that clinicians regularly use in clinical practice to decide on a recanalization procedure, especially for MT.

Interestingly, the clinical characteristics of patients with acute symptomatic seizures in both of our case-controlled analyses did not significantly differ from matched controls for most parameters, even though we did not explicitly match for most of them. Patients with seizures in the IVT+MT+ group were more likely to have diabetes mellitus; this is most likely due to the smaller sample size that allows for significant differences in the non-matched parameters. In an earlier study, diabetes mellitus was not described as a risk factor for acute symptomatic seizures in multivariate analysis^5^. Patients with seizures treated with IVT and MT had a longer length of stay on stroke unit; this argues against a relative underestimation of seizures compared to the IVT-only control group.

Our results support the findings of several smaller studies that did not find an increased number of acute symptomatic seizures in patients undergoing IVT^26,28–30,32^. Some of these studies presented more clinical details of patients suffering from acute symptomatic seizures, but the overall numbers of patients with seizures were limited in most studies, ranging from n = 3 to n = 25. Several studies were also not able to match patients due to the limited patient numbers^26,30^. We therefore suggest that our results, although clinically less descriptive, still support the idea that the risk of acute symptomatic seizures is similar between those with and without recanalization efforts, i.e. IVT and MT (in conjunction with IVT).

Reperfusion injury has been proposed as a mechanism of recanalization-related seizure risk^23^. Due to the average reperfused territory being larger after successful MT than in most thrombolysed patients, seizure risk should be relatively higher. To our knowledge, only one study evaluated the relative acute seizure risk in patients undergoing MT^24^, and one other estimated the relative post-stroke epilepsy risk^23^. Brigo et al.^24^ did not find a higher number of MTs in patients with seizures, but Naylor et al.^23^ found a higher risk of post-stroke epilepsy. Our results are congruent with those of Brigo et al.^24^; however, post-stroke epilepsy could not be evaluated in our database due to a lack of long-term follow-up. Further limitations of our study include the lack of several parameters that have been shown to influence the risk of acute symptomatic seizures such as a cortical stroke location and hemorrhagic transformation^36^. Additionally, we have no information regarding pre-existing epilepsy or seizures in the pre-hospital phase, and the exact timing of the seizures was not reflected in the database. A further possible point of criticism is that not all patients who received a recanalization procedure could be assigned to a control in the matching group, especially younger patients with more severe strokes. However, we believe that this does not impair the reliability of the results. Based on clinical judgment, it is plausible that younger and more severely affected patients could not be matched in our database, as therapy would be more often tried in these patients and thus less non-treated patients are available. We tried to mitigate this by not comparing patients with MT to those without any recanalization therapy alone but rather those with IVT and MT to a historical cohort of those with only IVT in the “era” before MT became available. The major strengths of our analyses are the population-based approach with mandatory inclusion of all stroke cases in a defined population of more than 6 million, the high overall patient number (135,117 adult patients) with acute ischemic strokes, and a sufficient number of patients with acute symptomatic seizures that allowed for sound statistical comparisons.

In summary, no statistically significant increases in the frequency of acute symptomatic seizures were found in patients with ischemic stroke and either IVT or additional MT treatment compared to controls.

## Data Availability

Data are available upon request by qualified researchers from the Geschäftsstelle Qualitätssicherung Hessen (GQH), pending certain prerequisites.

## Code availability

All code used is available as part of SPSS, version 26 (IBM Corp., Armonk, NY, USA).

## References

[1] Bladin CF (2000). Seizures after stroke. Arch. Neurol..

[2] Brondani R (2019). Risk factors for epilepsy after thrombolysis for ischemic stroke: a cohort study. Front. Neurol..

[3] Couillard P (2012). Subacute seizure incidence in thrombolysis-treated ischemic stroke patients. Neurocrit. Care.

[4] Jung S (2012). Adverse effect of early epileptic seizures in patients receiving endovascular therapy for acute stroke. Stroke.

[5] Zöllner JP (2020). National institutes of health stroke scale (NIHSS) on admission predicts acute symptomatic seizure risk in ischemic stroke: a population-based study involving 135,117 cases. Sci. Rep..

[6] Lamy C (2003). Early and late seizures after cryptogenic ischemic stroke in young adults. Neurology.

[7] Szaflarski JP (2008). Incidence of seizures in the acute phase of stroke: a population-based study. Epilepsia.

[8] Reith J, Jorgensen HS, Nakayama H, Raaschou HO, Olsen TS (1997). Seizures in acute stroke: predictors and prognostic significance. the copenhagen stroke study. Stroke.

[9] Krakow K (2010). Predictors of acute poststroke seizures. Cerebrovasc. Dis..

[10] Strzelczyk A (2010). Prospective evaluation of a post-stroke epilepsy risk scale. J. Neurol..

[11] Siebenbrodt K (2020). Recommendation for a definition of acute symptomatic seizure. Nervenarzt.

[12] Beghi E (2010). Recommendation for a definition of acute symptomatic seizure. Epilepsia.

[13] Camilo O, Goldstein LB (2004). Seizures and epilepsy after ischemic stroke. Stroke.

[14] Forsgren L, Bucht G, Eriksson S, Bergmark L (1996). Incidence and clinical characterization of unprovoked seizures in adults: a prospective population-based study. Epilepsia.

[15] Menon B, Shorvon SD (2009). Ischaemic stroke in adults and epilepsy. Epilepsy Res..

[16] Hacke W (2008). Thrombolysis with alteplase 3 to 4.5 hours after acute ischemic stroke. N. Engl. J. Med..

[17] Hacke W (2004). Association of outcome with early stroke treatment: pooled analysis of ATLANTIS, ECASS, and NINDS rt-Pa stroke trials. Lancet.

[18] Wahlgren N (2007). Thrombolysis with alteplase for acute ischaemic stroke in the safe implementation of thrombolysis in stroke-monitoring study (SITS-MOST): an observational study. Lancet.

[19] Wahlgren N (2008). Multivariable analysis of outcome predictors and adjustment of main outcome results to baseline data profile in randomized controlled trials: safe implementation of thrombolysis in stroke-monitoring study (SITS-MOST). Stroke.

[20] Saver JL (2015). Stent-retriever thrombectomy after intravenous t-Pa versus t-Pa alone in stroke. N. Engl. J. Med..

[21] Campbell BC (2015). Endovascular therapy for ischemic stroke with perfusion-imaging selection. N. Engl. J. Med..

[22] Berkhemer OA (2015). A randomized trial of intraarterial treatment for acute ischemic stroke. N. Engl. J. Med..

[23] Naylor J (2018). Association between different acute stroke therapies and development of post stroke seizures. BMC Neurol..

[24] Brigo F (2020). Intravenous thrombolysis with tpa and cortical involvement increase the risk of early poststroke seizures: results of a case-control study. Epilepsy Behav..

[25] Alvarez V, Rossetti AO, Papavasileiou V, Michel P (2013). Acute seizures in acute ischemic stroke: does thrombolysis have a role to play?. J. Neurol..

[26] De Reuck J, Van Maele G (2010). Acute ischemic stroke treatment and the occurrence of seizures. Clin. Neurol. Neurosurg..

[27] Keller L, Hobohm C, Zeynalova S, Classen J, Baum P (2015). Does treatment with t-Pa increase the risk of developing epilepsy after stroke?. J. Neurol..

[28] Bentes C (2017). Epileptic manifestations in stroke patients treated with intravenous alteplase. Eur. J. Neurol..

[29] Castro-Apolo R, Huang JF, Vinan-Vega M, Tatum WO (2018). Outcome and predictive factors in post-stroke seizures: a retrospective case-control study. Seizure.

[30] Nesselroth D, Gilad R, Namneh M, Avishay S, Eilam A (2018). Estimation of seizures prevalence in ischemic strokes after thrombolytic therapy. Seizure.

[31] Anadani M (2019). Incidence, predictors, and outcome of early seizures after mechanical thrombectomy. J. Neurol. Sci..

[32] Belcastro V (2020). Incidence of early poststroke seizures during reperfusion therapies in patients with acute ischemic stroke: an observational prospective study: (TESI study: “trombolisi/trombectomia e crisi epilettiche precoci nello stroke ischemico”). Epilepsy Behav..

[33] Eriksson H (2020). Acute symptomatic seizures and epilepsy after mechanical thrombectomy. Epilepsy Behav..

[34] Zöllner JP (2020). Acute symptomatic seizures in intracerebral and subarachnoid hemorrhage: a population study of 19,331 patients. Epilepsy Res..

[35] Bentes C (2017). Post-stroke seizures are clinically underestimated. J. Neurol..

[36] Thevathasan A (2018). Association between hemorrhagic transformation after endovascular therapy and poststroke seizures. Epilepsia.

